# A qualitative study exploring access to online hearing loss information and support for adults with hearing loss

**DOI:** 10.3389/fdgth.2026.1692717

**Published:** 2026-06-23

**Authors:** Alicia Zou, Tsz Yui Wong, Jessica Turner, Diana Tang, Duncan Meldrum, Elizabeth Davies, Kate Sheng, Simon O’Toole, Jane Lee, Liz Gill, Melanie Ferguson, Bamini Gopinath

**Affiliations:** 1 Macquarie University Hearing Research Centre, Faculty of Medicine, Health and Human Sciences, Macquarie University, Sydney, NSW, Australia; 2 Australian Astronomical Optics, Macquarie University, Sydney, NSW, Australia; 3 Deafness Forum Australia, Canberra, ACT, Australia; 4 John Walsh Centre for Rehabilitation Research, Sydney, NSW, Australia; 5 Kolling Institute, Faculty of Medicine and Health, The University of Sydney, Sydney, NSW, Australia; 6 School of Allied Health, Curtin University, Perth, WA, Australia

**Keywords:** adults, behaviour change, eHealth, hearing loss, qualitative, theoretical domains framework

## Abstract

**Introduction:**

This qualitative study explores the barriers and facilitators experienced by adults with hearing loss in accessing hearing-related information and services. Findings were interpreted using the Theoretical Domains Framework, which will be used to inform the design of a novel consumer-centred website—Hear4Health.

**Methods:**

A total of 13 participants (19–78 years) were recruited. Nine consumers with hearing loss participated in the focus groups, four of whom also served as representatives of consumer organisations. 12 of the 13 participants were subsequently interviewed (51.25 ± 20.35 years), including seven consumers, two consumer organisation representatives, and three who fulfilled dual roles.

**Results:**

Nine themes emerged from the interviews under seven theoretical domains: knowledge, environmental context and resources, social influence, beliefs about capabilities, beliefs about consequences, social/professional role and identity, and behavioural regulation. Barriers to access included the themes poor awareness of hearing loss, low-quality information, mistrust in the hearing industry, stigma, unrealistic expectations for hearing technologies, and deaf identity. Facilitators identified included peer support and the value of lived experience, informed decision-making, and self-efficacy.

**Discussion:**

These findings provide crucial insights for the development of Hear4Health. Grounded in the Theoretical Domains Framework, this research underscores the importance of addressing both individual and systemic factors to improve digital access to hearing healthcare and empower adults with hearing loss to make confident and informed choices.

## Introduction

Hearing loss is the third leading cause for years lived with a disability and a rising global public health issue (1). Despite the availability of effective interventions, adults with hearing loss delay seeking help for an average of 8.9 years (2). This delay may be attributed to several barriers, including cost, limited accessibility of audiological services, and a lack of understanding of hearing services and pathways. Early identification and effective management of hearing loss is crucial as auditory deprivation, when left untreated, can lead to negative effects on communication, social participation, mental and cognitive health, and overall quality of life (3–8).

In this digital age, online health information-seeking has become increasingly prevalent. Online health information-seeking holds significant potential to empower individuals by improving their understanding of health concerns and supporting informed decision-making for effective condition management. However, the success of online health information-seeking and whether it increases the likelihood of engaging with a health professional may be impacted by individual perceptions of the credibility and accuracy of the online information (9–11).

This relationship is further influenced by an individual's level of health literacy, which can be defined by “the degree to which individuals can obtain, process, understand, and communicate about health-related information needed to make informed health decisions” (12, 13). Individuals with hearing loss often face unique challenges in accessing health-related information, including communication challenges with medical professionals and poorly tailored resources (14–16). These barriers may also contribute to the lower health literacy levels among adults with hearing loss, as well as poorer health outcomes (16–19). Furthermore, the research demonstrates that hearing-related information, both online and offline, is often of poor quality and presented at a level that is too complex for the average consumer (20, 21). Yet, online education has demonstrated potential in improving both overall and hearing-specific health literacy among people with hearing loss (16). Ultimately, these findings highlight the importance of delivering trustworthy, high-quality, and consumer-appropriate sources of hearing health information and resources to better meet the unique needs of adults with hearing loss and promote earlier help-seeking and greater adoption of hearing technologies.

In Australia, there is currently no single, comprehensive online reference point for consumers to access unbiased and authoritative hearing health information on prevention through to management of hearing loss. To address this, current efforts are underway to co-design Hear4Health, an online platform to support adults with hearing loss in Australia (22). This platform will be a website that provides access to reliable, consumer-friendly information to support informed decision-making, improve transparency across the hearing health journey, and encourage the adoption of healthy hearing behaviours (22).

To inform the development of this website, we conducted a series of focus groups and follow-up semi-structured interviews with adults with hearing loss and consumer group representatives. This study aims to identify the unmet needs and challenges consumers face in accessing hearing health information and support. The qualitative data were deductively analysed under the Theoretical Domains Framework (TDF) to identify opportunities for behaviour change interventions that could improve access to and engagement with hearing healthcare through an online resource such as Hear4Health (23).

## Materials and methods

### Ethical approval

This study was approved by the Macquarie University Human Research Ethics Committee (ID: 16744) on the 20th June 2024.

### Study design

This was a qualitative study involving focus groups and semi-structured interviews with consumers with hearing loss and consumer organisation representatives.

A qualitative approach was adopted in current study to allow depth and flexibility in exploring participants' lived experiences with hearing loss, their interactions with management options, and their use of information resources. More importantly, this study forms part of the co-design process within the Hear4Health project where consumers' inputs directly inform the following stages of intervention development.

### Participant recruitment

Participants, including consumers and hearing health representatives from Deafness Forum Australia, were recruited internally through Deafness Forum Australia's consumer networks and member organisations (via email, social media, and newsletter). Deafness Forum Australia is the national peak body representing Australians who are deaf and hard of hearing. Consumers were eligible to participate if they were aged 18 years or older, had a clinician-verified hearing loss, and proficient in English. Representatives of Deafness Forum Australia were eligible if they were aged 18 years or older, active members of the organisation, and proficient in English. Interested individuals were screened by the research team and eligible participants were selected based on the principles of stratified purposeful sampling, using the key variables of age, gender, hearing device use, hearing loss cause, and geographical location, to ensure that a diverse range of rich perspectives were captured in the data (24). All eligible participants were invited to join the study's Advisory Group and were provided with a Terms of Reference outlining member responsibilities to read and sign. Before taking part in focus groups and interviews, Advisory Group members also received a Participant Information and Consent Form to read; informed verbal consent was obtained for both participation and audio recording prior to the commencement of focus group or interviews.

Heterogeneous sampling was adopted in the current study to identify both shared themes across adults with hearing loss and perspectives that may be specific to particular subgroups (e.g., perspectives that are unique to age-related hearing loss specifically). This approach allows a comprehensive understanding of various user needs and experiences, and effectively informs the development of an inclusive and broadly accessible digital resource.

### Focus group protocol

Participants attended one of two focus groups conducted in September 2024. The first was held online via Microsoft Teams with interstate participants, while the second took place in person at Macquarie University and was attended by local participants. A focus group guide was developed to explore participants' experiences accessing hearing health information and resources, with a particular focus on identifying key barriers and facilitators to accessing online information (Supplementary Table S1). The questions were developed iteratively through collaboration between the research and user experience (UX) design teams to ensure that existing gaps and challenges were adequately captured to inform the design of Hear4Health. Prior to implementation, the guide was reviewed and tested by a member of the research team with lived experience of hearing loss to confirm its relevance and appropriateness. Each focus group proceeded for two hours and was audio-recorded to capture the full discussion. Recordings were transcribed verbatim using Microsoft Teams or Microsoft Word, and key insights were identified through a preliminary review of the transcripts.

### Semi-structured interview protocol

Following the focus groups, semi-structured one-on-one interviews were conducted either in-person at Macquarie University or online via Microsoft Teams by one of two researchers (AZ, DT) between October and November 2024. This flexible yet guided approach ensured consistency across interviews while still allowing room for participants to express and elaborate on their unique experiences, thereby contributing to a rich and high-quality dataset (25). An interview guide drawing on the key themes that had emerged from the focus groups was collaboratively developed and progressively refined by the research and UX teams.

Questions were designed to explore participants' experiences with hearing loss and the management options offered to them along with their use of hearing loss information and resources both online and offline. Preferred topics and information formats, as well as perceptions of how hearing loss is represented publicly were also included (Supplementary Table S2). The questions were targeted to identify specific barriers and facilitators to accessing hearing health information, services, and management, with the goal of informing the design of the Hear4Health platform. The interview guide was piloted once within the research team to affirm clarity and comprehensiveness.

Interviews took place between October and November 2024. Interviews were audio-recorded and transcribed verbatim using Microsoft Teams or Microsoft Word and checked by a researcher (AZ) for accuracy. Transcripts were shared with participants for review before being de-identified and finalized. Demographic information was collected during the interviews and included age, gender, type of hearing loss (unilateral/bilateral, severity if known), hearing device use (e.g., hearing aids, cochlear implants), nationality, languages spoken, treatments sought, and other relevant characteristics.

### Data analysis

Finalised transcripts were imported into NVivo 14 (QSR International, Australia) for qualitative analysis. Interview data were deductively analysed under the TDF, which is a framework commonly used to understand influences on health-related behaviours by grouping them into theoretical domains such as knowledge, social influence, and environmental context and resources to identify opportunities for behaviour change (26) Therefore, the qualitative data were systematically coded into all relevant domains and categorised as either a “barrier” or “facilitator”. Within each domain, inductive coding was then conducted to identify overarching themes.

Two researchers (AZ, JT) independently coded the transcripts, then met to discuss and reach consensus on the assigned domains. All data were subsequently revisited and recoded by both researchers to support analytic reflexivity. The inter-rater reliability was very strong indicating high agreement between coders, with an overall unweighted Cohen's kappa coefficient of 0.94, where a coefficient of 1.0 indicates complete agreement. Themes were then generated by one researcher, reviewed and agreed upon by the second, and further refined through discussion with the principal investigator.

## Results

### Participants

A total of 13 participants (aged 19 to 78 years) were recruited between June and August 2024. Nine consumers with hearing loss participated in the focus groups, four of whom also served as representatives of Deafness Forum Australia. 12 of the 13 participants were subsequently interviewed (5 female; mean age = 51.25, SD = 20.35) (Table 1). One focus group participant was unavailable to participate in a follow-up interview. Interviews ranged in duration from 21 to 85 min, with an average length of 48 min. Among those interviewed, seven were consumers only, two were Deafness Forum representatives only, and three held dual roles as both consumers and representatives (Table 1). Of the participating consumers, one had no history of hearing device use, two were bimodal users (hearing aid and cochlear implant), four were bilateral cochlear implant users, and three were hearing aid users (unilateral or bilateral) (Table 2). Of the hearing aid users, only one reported consistent and current use. 70% of participants reported acquired hearing loss, with age-related (*n* = 3) being the most common cause of hearing loss observed in participants, in addition to less common causes such as hearing loss due to infection and tumour (Table 2).

**Table 1:** Demographic characteristics of interview participants (*n* = 12).

Characteristic	Mean (SD)	Range
Age	51.25 (20.35)	19–78

	*N*	%
Gender		
Male	7	58.33
Female	5	41.67
Participant role		
Consumer only	7	58.33
Representative only	2	16.67
Consumer AND Representative	3	25
Employment status		
Employed	6	50
Semi-employed	2	16.67
Retired	2	16.67
Unemployed	1	8.33
Volunteer	1	8.33
State/territory		
NSW	6	50
QLD	2	16.67
VIC	1	8.33
WA	1	8.33
NT	1	8.33
ACT	1	8.33

**Table 2:** Hearing-related characteristics of interview consumers (*n* = 10).

Characteristic	*N*	%
Device use		
Unilateral or bilateral hearing aids	4	40
Unilateral or bilateral cochlear implants	4	40
Bimodal	1	10
None	1	10
Hearing loss cause		
Age-related	3	30
Congenital	2	20
Infection	2	20
Tumour	1	10
Noise-related	1	10
Unknown	1	10

### Focus group findings

Focus group discussions were separated into three sections as per the question guide: (1) consumer experiences with hearing loss and the hearing healthcare pathway, (2) consumer experiences with existing online hearing health information and resources, and (3) evaluation of existing hearing health media types and content (Supplementary Table S1).

#### Consumer experiences with hearing loss and the hearing healthcare pathway

Participants expressed experiencing gaps in both support and information. Consumers identified having limited access to clear information about referral processes for hearing loss and the costs associated with hearing tests and services. A common concern amongst participants was that hearing service providers could not be trusted due to their perceived commercial motivations, with several preferring to seek help from independent audiologists. Participants also noted a gap in consumer support beyond their initial diagnosis or management, as well as a need for audiologists to set more realistic expectations for hearing device (including hearing aids) outcomes. Perceived stigma surrounding hearing aid use was also raised as a barrier to help-seeking.

#### Consumer experiences with existing online hearing health information and resources

Consumers expressed a strong desire for accessible, comprehensive, and consumer-friendly content that is neutral and free from commercial bias, with a call for a centralised online directory featuring credible information, resources, and a listing of healthcare professionals and their services. Consumers requested comprehensive information targeting individuals with hearing loss as well as their communication partners that covers a wide range of topics, including hearing technologies, lifestyle and workplace accommodations, support services, and self-advocacy. Participants also discussed the need to engage a more diverse audience to promote prevention and early intervention of hearing loss. Many consumers expressed an interest in connecting with others through online support groups to share lived experiences and practical advice, especially regarding the use of hearing technologies (such as hearing aids and assistive listening devices).

#### Existing hearing health media

Participants called for improved marketing and community messaging around hearing health and hearing technologies. Current marketing strategies were perceived by participants as failing to connect to a diverse audience or raise meaningful awareness of hearing loss. Participants believed that media campaigns should go well beyond promoting hearing testing services focused on the provision of a hearing aid, and to better educate the community about hearing loss and the full range of available hearing solutions, including cochlear implants, assistive listening devices, and lifestyle accommodations.

### Interview themes

**Figure 1:**
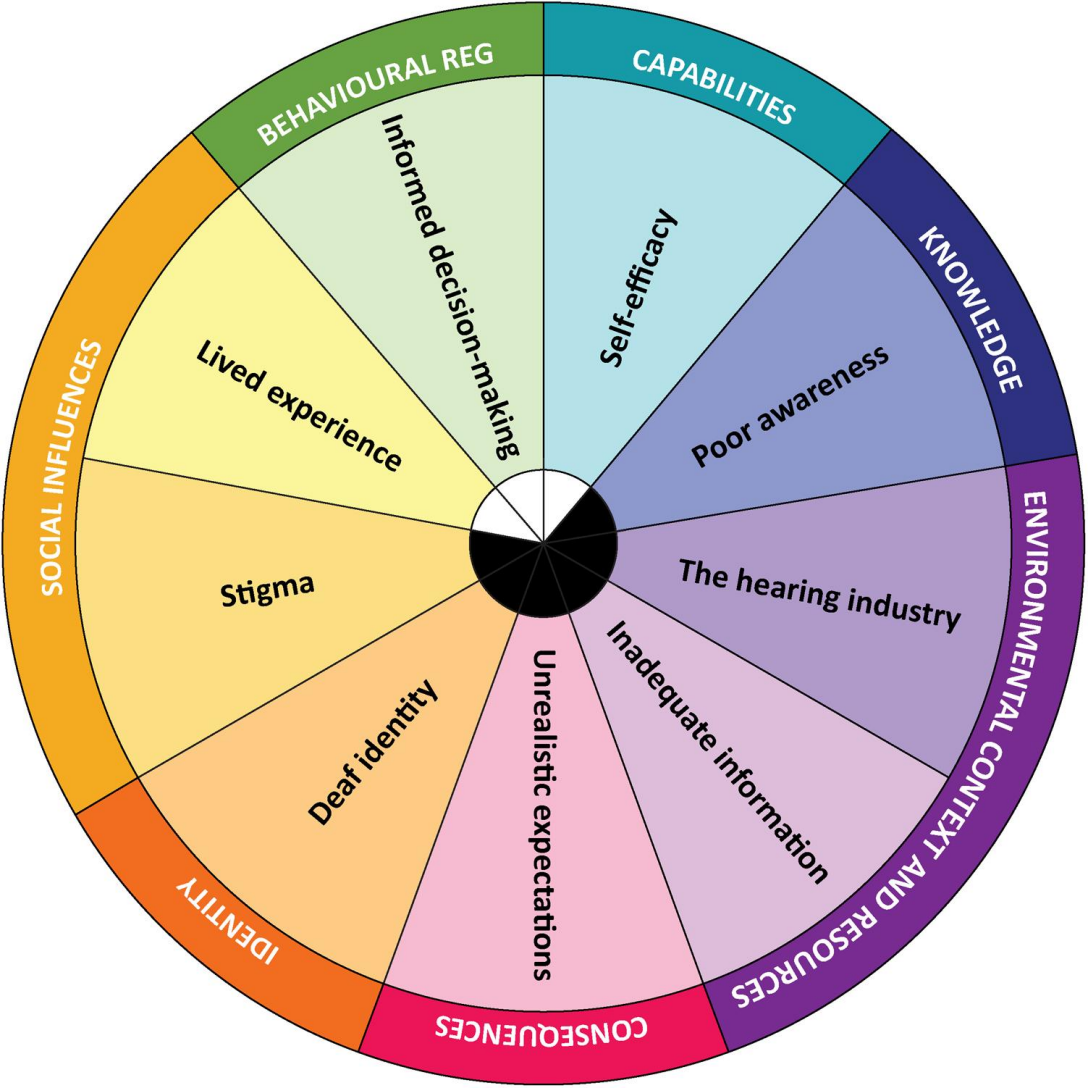
Barriers and facilitators to the access of hearing healthcare interpreted under the Theoretical Domains Framework. Interview data was deductively coded to the Theoretical Domains Framework. Themes emerged from seven theoretical domains and were categorised as either a barrier or facilitator to the access of hearing healthcare. Barriers included poor awareness of hearing loss, the hearing industry, inadequacy of hearing health information and resources, unrealistic expectations for hearing technology, deaf identity, and stigma. Facilitators included the value of lived experiences, informed decision-making, and self-efficacy. Key: inner ring—barrier (black)/facilitator (white), middle ring—theme, outer ring—theoretical domain.

A total of nine key themes emerged from the interviews, mapped across seven TDF domains: knowledge, environmental context and resources, social influences, behavioural regulation, beliefs about consequences, beliefs about capabilities, and social/professional role and identity. Of these themes, six were identified as barriers and three as facilitators to the effective management of hearing loss (Figure 1). Barriers included poor awareness of hearing loss, the hearing industry, inadequacy of existing hearing health information and resources, stigma, unrealistic expectations for hearing technology, and deaf identity. Facilitators included informed decision making, self-efficacy, and the value of lived experience.

#### Barriers

##### Domain: knowledge

###### Theme: poor awareness of hearing loss

Limited knowledge of hearing loss was identified as one of the most prominent barriers to accessing quality hearing healthcare. A key belief expressed by participants was that the community lacks awareness and understanding of hearing loss, perhaps due to insufficient education. One consumer (03C) noted, “*there needs to be more general public education about hearing loss*”, with another (05C) stating, “*I think a lot of people don't have an understanding of deafness or how to communicate with a person with hearing loss*”.

Another common sentiment among participants was the lack of awareness concerning the full range of hearing supports available to them beyond hearing aids. One consumer (12C) commented, “*I just thought it was hearing aids or nothing*”, while another (02C) remarked, “*the general community thought that if you had a hearing aid, you'd hear fine*”. Participants expressed a desire to know more about various aspects of hearing loss and its management, including available treatment options, accommodations, environmental adaptations, support technologies, support groups, and mental health resources.

“I think there should just be more information available and accessible regarding … assistive [listening] devices and … how you can adapt your environment and like, I guess, just the options that are available in that sense to, yeah, make people hear better in their day to day lives rather than solely relying on a hearing aid cause it's more than that and I didn't realise”. (12C)

##### Domain: environmental context and resources

Two themes emerged as barriers to effective hearing loss management within the domain of *environmental context and resources*.

###### Theme: the hearing industry

Two themes emerged as barriers to effective hearing loss management within the domain of *environmental context and resources*. The first relates to the hearing industry itself, primarily participants' perceptions of overcommercialisation and the problematic advertising of hearing aids, with little regard for improving hearing health.

“Some of the ads on television are just appalling. I mean, because they're about selling technology. They're not about addressing hearing loss”. (02C)

“I have yet to see one [ad] that's actually honest and factual”. (01C)

“Most of them were very much focused on consumers who would be candidates for hearing aids … And so for me, you know, that was problematic”. (02C)

“To me, it's still very car salesy-like, which I don't like. And I think that that makes people very sceptical and concerned about where to go and what to do. (10R)

“It's sort of unfortunate that it's all become very commercial”. (03C)

Most participants also expressed concerns over bias in available hearing loss-related information towards hearing aid manufacturers. One consumer (05C) stated, “*I think the biggest problem is getting impartial opinion*”, while another (02C) observed, “*there's no industry controls at the moment around standards of information that's being pushed out in this industry*”.

The influence of commercialisation within the hearing industry was also observed in some participant's interactions with hearing service professionals. Several described feeling pressured to adopt specific hearing solutions, with one participant (04C) conveying, “*clearly, he wanted to sell me hearing aids*”. Similarly, another consumer (05C) noted, “*my audiologist didn't want me to go from a hearing aid to a cochlear implant*”, with the reasoning, “*I think it would make him lose his money*”. In reference to free hearing tests offered by particular hearing clinics, one participant (012C) mentioned, “*they just seem like they're trying to sell you something and I don't feel like they are very reputable*”.

###### Theme: inadequacy of existing hearing health information and resources

The second theme under *environmental context and resources* captured participants' concerns that existing hearing health information is generally inadequate, described as low quality, fragmented, outdated, and unreliable. As one participant (03C) explained, “*accessing is the easy part but whether the information you're getting is what you want*”. Other common sentiments from participants included*, “it's almost like there's a bit too much information or it's too dispersed* (09C)”, “*there's so much misinformation out there as well, like, even if it did say something, I would be really hesitant to believe it* (06C)”, and finally, “*a lot of it is not evidence based* (02C)”.

Several participants also perceived that a lot of the available hearing loss-related information was not written in a consumer-friendly manner or tailored to consumer needs. One consumer (06C) describes it as “*not relevant, too technical*”, while another (02C) proposed, “*we need a complete review of information so that it becomes consumer focused. Consumer driven*”.

In addition to concerns around information, some participants also voiced feeling unsupported by current hearing healthcare systems and policies. For example, in reference to post-cochlear implantation care, one consumer (05C) remarked, “*I just felt it would've been good but they didn't tell me and I felt that they didn't give me much support on it as well*”. A Deafness Forum representative (07R) also raised issues with government-run funding schemes: “*there is often a lack of clarity when it comes to some of, like, these regulatory bodies and the sort of rules that can be quite arbitrary*”, making it difficult for consumers to navigate.

##### Domain: social influence

###### Theme: stigma

A strong consensus emerged among participants that hearing loss, as a disability, is still widely perceived negatively by the general public. One consumer (05C) observed, “*there are still some issues, I feel, within the community with the attitude towards people with disability*”. The workplace was identified as a setting where discrimination is frequently experienced. One consumer (09C) revealed, “*I got sacked by my employer for being deaf*”, while another (05C) shared that their employer “*wanted to sack me because I was hearing impaired*”.

Participants acknowledged that the stigma was associated with the visibility of hearing devices such as hearing aids. For some, this influenced their decisions around treatment. One consumer (03C) explained, “*when I was that age* *…* *I didn't want to look like I was wearing hearing aids, like, so rather than being so concerned about trying to get perfect hearing* *….* *I didn't wanna look like I was wearing hearing aids*”. Other participants agreed that it was the visibility of hearing devices that contributed to the stigma of hearing loss.

“Stigma, to some extent, only comes out because you can see the device. Otherwise, deafness is essentially like an invisible disability to some extent, right?” (07R)

“Look there's all this thing about, oh, stigma of hearing loss, etcetera. And I'm thinking, I mean, really? Still? Now? But I get it 15, 20 years ago, you had these big pink things hanging out your ears, squealing away … I meet some people; I've talked to them. They've got no idea because, you know, it's hidden behind my ear”. (08C)

Many participants attributed community misperceptions about hearing loss to the media, specifically, the limited portrayal of positive deaf and hard-of-hearing identities. One consumer (08C) observed, “*there's very little about hearing loss in the media in Australia*”. Another consumer (06C) agreed, saying, “*there's not too much portrayal of deaf identities and hearing health and stuff in the media as it is, but if there was, I feel like it'd be pretty negative*”.

Participants emphasised the persistent association between hearing loss and older age. One representative (10R) noted, “*I still think that it's portrayed as an old person disability when it's not, we know that*”, with a consumer (12C) echoing, “*as for, like, hearing loss in the media, I haven't really seen too much and* *…* *if I have, they're more trying to appeal to an older audience, for, I guess, age-related hearing loss. You never really see any younger people in those kinds of campaigns*”.

Participants also reflected on how the language used to describe hearing loss contributes to stigma. Some found that certain terms reinforced negative perceptions of hearing loss and hearing technology:

“I don't like this word [hearing] “aid”. It has connotations that I think don't help the image of the product either”. (02C)

“I think you can really feel the stigma based on the way that they describe the disability”. (06C)

“But I think there's always going to be stigma as long as you know it's still going to be framed as a loss, as a disability, as an impairment”. (07R)

##### Domain: beliefs about consequences

###### Theme: unrealistic expectations for hearing technology

This theme falls beneath the domain of *beliefs about consequences* and reflects participants' perceptions that existing information and advertising frequently sets unrealistic and inflated expectations for the effectiveness of hearing technologies, in particular, hearing aids. One consumer (02C) noted, “*a lot of the information didn't help people establish good expectations about what the technology* *…* *could do to support them*”. Several consumers expressed that they believed hearing aids would return their hearing to “normal”, often comparing them to how glasses can successfully restore vision.

“I thought at that time they would fix things. I mean, I had the view that because I wore multifocal glasses and they sort of made my sight perfect, I thought that these would make my hearing perfect … And they weren't”. (03C)

“They're not like glasses. You know, you put your glasses on, you automatically see better, you know”. (09C)

“It's not like glasses where you put [them] on and go “wow”. It takes time”. (10R)

One representative (07R) describes the harm that can result from exaggerated advertising and promising false hope to consumers:

“I find if it comes to industry advertising of hearing aids, they are just so over the top, like … this hearing aid can just do everything, and it's just going to cure and solve every problem … it's dishonest like that. And I believe that that leads to very significant long-term damage because then that person is likely to then just put their hearing aid in the bedside table”.

One consumer (01C) corroborated the significant consequences of such unmet expectations, sharing, “*I had stopped wearing hearing aids a little bit before that because they didn't -They weren't working. My mental health was through the floor*”.

To address this issue, many participants stressed the need for more accurate and transparent information about the limitations of hearing technologies for consumers to be able to establish realistic expectations.

“If the information was clear about what the limitations are, when they're likely to work, when they're not likely to work, etc. If that information was there, then people's expectations will become more realistic”. (02C)

“Yeah, I think some more realistic expectations for what the hearing aids could do for me”. (12C)

##### Domain: social/professional role and identity

###### Theme: deaf identity

The final barrier identified from the interviews relates to the theme of deaf identity, coded to the domain of *social/professional role and identity*. Some participants expressed uncertainty about where they fit within the broader deaf or hard-of-hearing community. One consumer (04C) reflected, “*I'm not sure that I'm sort of deaf enough*”, adding, “*the issues that I have as someone older deteriorating [is] obviously going to be a hell of a lot different from a young person who was born with hearing loss or has an accident or has an illness*”.

Other participants acknowledged this spectrum of hearing loss, with one consumer (09C) referring to “*people who were more experienced deaf people than I am*”. This consumer also went on to describe the emotional impact of not feeling a sense of belonging:

“I'm just really stuck, not fitting in anywhere at the moment, so it's, you know, it's taken a really big psychological toll”.

In contrast, a consumer (06C) who has been deaf from birth highlighted that struggles with deaf identity may not be tied to level of “experience” or years lived with disability. He disclosed*, “I think that's a really complicated thing for me personally since I grew up with a hearing loss* *….* *I just didn't wanna know anything about it* *…* *And, you know, struggling with my deaf identity as well, I was just trying to like really run the other way, you know what I mean? I didn't want to accept that I had a hearing loss*”. The diversity of deaf identities experiences was acknowledged by one representative, who observed, “*most people fall into one of these two camps, like you either want to hide it, you want to minimise the stigma, you want to minimise the display of the hearing loss or you want to express it loud and proud*”.

#### Facilitators

Three themes emerged as facilitators of successful hearing loss management from the interviews, each underpinned by a different theoretical domain: *social influence, behavioural regulation, and beliefs about capabilities*. Conceptually, there was considerable overlap between the themes of *informed decision-making* (behavioural regulation) (26) and *self-efficacy* (beliefs about capabilities) (27), as both relate to the adoption and maintenance of healthy hearing behaviours. As a distinction, *informed decision-making* refers to the role of informational resources and decision-support tools in helping consumers make appropriate choices about their hearing health. *Self-efficacy*, on the other hand, refers to an individual's belief in their own ability to successfully bring about desired results, and can be achieved by equipping individuals with the knowledge, skills, and confidence to take control of and actively manage their hearing loss in day-to-day life.

##### Domain: behavioural regulation

###### Theme: informed decision-making

*Behavioural regulation*, as defined by Atkins et al. (28), refers to “anything aimed at managing or changing objectively observed or measured actions” (28). Participants identified that actively participating in their hearing healthcare through informed decision-making contributed improved outcomes, with one consumer (06C) stating, “*they [hearing healthcare professional] teach me what's happening and stuff because I'm interested now. And, you know, they'll let me map [my cochlear implants] myself as well, you know, switch some levels up. And I think that it leads to a much better outcome when I'm a lot more involved*”.

Several participants recognised the importance of consumer-focused information and tools that enable individuals to make the most appropriate choices and tailor their hearing care to their unique needs. One consumer (03C) suggested, “*I just feel that there's a place for an industry body whether it's government sponsored or whatever. And it could be similar to the business model that Choice magazine use, where there can be independent assessment, not only of the various devices and the ways of measuring individuals hearing profiles but also if, you know, if there's some something new comes up*”. Another consumer (12C) reflected, “*I would have liked to also know what options there were for me and kind of evaluate for myself what would have worked best*”. One consumer (01C) advocated for gentle, proactive educational messaging to reinforce positive hearing behaviours: “*if you have a hearing loss, you still have to wear your hearing aids. Even if you think they're not working for you*”.

A common notion expressed among participants was the need for a more holistic approach to hearing healthcare, beyond just hearing aids. Participants expressed interest in information about environmental adaptions, using assistive technology and accommodations, as well as applying lifestyle-based strategies to facilitate improved hearing care. One consumer (12C) was interested in information about “[assistive listening] *devices and how you can adapt your environment* *…* *just the options that are available in that sense to, yeah, make people hear better in their day-to-day lives rather than solely relying on a hearing aid, because it's more than that and I didn't realise*”. One representative (07R) similarly described, “*what would be really refreshing for me would be like if someone had a vignette and they say like, oh, you know what really helps me in noisy environments? Lip reading. Or like, you know, I really value having, being aware of the environment and situating and placing myself in the place that's going to be the best for my listening. And you know, something that's going to decentre it from, like the device aspect as well*”. There were also calls to “*focus on the technology to improve quality of life (02C)*”, with another consumer accepting that “*I'm not necessarily going to improve my hearing, but [assistive technologies] might make my life easier (05C)*”. Lastly, one consumer (01C) raised the importance of promoting resources for mental health support: “*for me, instantly acknowledging that mental health is a significant part of this*”.

##### Domain: beliefs about capabilities

###### Theme: self-efficacy

The theme of self-efficacy emerged as a key facilitator to hearing loss management under the domain of *beliefs about capabilities*, which has been defined as the “acceptance of the truth, reality or validity about an ability, talent, or facility that a person can put to constructive use” (26).

Participants recognised the importance of giving individuals the skills and the confidence to take an active role in managing their hearing health. One consumer (02C) asserted, “[the information] *needs to also empower consumers and give them the power they need to be able to manage their hearing loss*”. This empowerment might take many forms, such as, “*managing the environment, giving people skills, giving people the confidence to say to someone, I'm sorry, I can't have a conversation with you in this environment (02C)*”. Another consumer (08C) highlighted that many of these strategies can be both simple and effective: “*there are some things you can do and then a lot of these things are simple, really simple, actually. Close the window, turn the radio down*”.

Self-advocacy, or the ability to communicate one's needs and seek appropriate support, was also recognized to be a major element of self-efficacy. However, participants acknowledged that advocating for oneself can be difficult. As one representative (07R) noted, “*there's lots of challenges when it comes to advocating for yourself*”. For one consumer (09C), external support was necessary: “*I was having a really hard time with my employer, I was* *…* *searching for deaf advocacy or disability advocacy, because I was fighting this huge* *…* *department on my own*”.

##### Domain: social influence

###### Theme: value of lived experience

The second theme within the domain of social influence emphasised the significant value participants placed on receiving information and support from others with lived experience of hearing loss. As one consumer (06C) observed, “*what everyone values is just having someone else who's been through it*”. For many, friends and peers served as primary sources of hearing-related information, as was the experience of one consumer (04C): “*to the extent I access hearing information, it would be my friends*”. In some cases, these social networks offered advice that had not been provided by hearing service professionals. One consumer (05C) recalled: “*I've never been suggested by any professional to go for a cochlear implant but a mate of mine, he had his done and he said, go for a cochlear implant, it's so much better than a hearing aid. And I tell you what, they are so much better than a hearing aid*”.

Other participants also described actively seeking out connection and support through deaf and hard-of-hearing community groups. One consumer (09C) shared, “*this actually comes back to* *…* *finding support. It's like trying to find [an] Auslan club or a deaf club that's near me. So that's sort of been one of the things that I've been searching for*”. Others turned to online platforms to connect with peers, including Facebook groups. As one consumer (05C) stated, “*also Facebook* *…* *is good for getting information from other deaf people. That's a very good way to get information*”.

Despite the perceived benefits of these consumer networks, one participant (03C) suggested that support groups may be underutilized due to a lack of their awareness:

“Whether the population at large, particularly older people like myself, I mean we wouldn't even know that there's such a support group exists—or I wouldn't have known—so more could be done to promote these types of groups and also just to let people know that it's really quite normal to have hearing problems as you get older”.

#### Domains reported less relevant

Interview transcripts were coded across all 14 domains, however, the nine distinct themes described above emerged from only seven domains. Some domains, including *goals* and *optimism*, were coded with similar frequency to more “relevant” domains such as *knowledge* or *social influences*, but were ultimately absorbed into other domains due to significant thematic overlap. For example, many quotes coded under *optimism* reflected pessimistic attitudes towards *the hearing industry* and were therefore integrated into this theme under *environmental context and resources*. Less relevant domains included *emotion*, *intentions*, *memory, attention, and decision processes*, *reinforcement*, and *skills*.

## Discussion

This qualitative study investigated the key barriers and enablers influencing access to hearing health information and support among adults with hearing loss, which will be used to inform the future development of an online resource—Hear4Health. Nine themes were identified to influence how consumers engage with hearing loss information and supports and mapped to seven key domains of the TDF (28). Six of these themes emerged as barriers and reflected both individual and systemic challenges, including limited community awareness of hearing loss, over-commercialisation within the hearing industry, a lack of trustworthy, evidence-based, and consumer-friendly information, hearing loss-related stigma, unrealistic expectations of hearing technologies, and the complex dynamics surrounding deaf identity. In contrast, three themes emerged as facilitators: improved access to transparent, comparative resources to support informed decision making; the development of self-efficacy through knowledge and skill building; and peer support from others with lived experience. Core ideas appeared across several themes, including the importance of holistic hearing loss care and the foundational roles of knowledge supported by high-quality information that facilitates healthy hearing behaviours. Combined, these themes underscore the multifaceted nature of hearing loss management and point to critical opportunities to improve consumer engagement with hearing loss information and resources through Hear4Health to aid enhanced hearing health and quality of life.

### Influences on poor hearing behaviours

Despite the increasing prevalence of hearing loss, especially among older adults, more than 60% of Australian adults with hearing loss do not receive any type of intervention (29). With known delays in hearing aid adoption by an average of 8.9 years (2), our findings contribute to the growing knowledge on barriers to help-seeking and highlight the complexity of factors driving this behaviour (26).

Notably, *poor community awareness around hearing l*o*ss* emerged as a foundational barrier under the domain of *knowledge*. This awareness is strongly governed by the nature of the *environmental context and resources* surrounding hearing loss, specifically the *inadequacy of available hearing health information*, another theme raised by participants. Without access to high-quality education and supporting resources around hearing loss, its early signs, and the importance of prompt intervention, individuals may struggle to recognise and acknowledge their hearing difficulties. This knowledge is crucial, as self-perception of hearing loss is a key facilitator of help-seeking behaviour and hearing aid adoption (30, 31).

Furthermore, participants also described the overcommercialisation of *the hearing industry* (environmental context and resources) to be a barrier to hearing loss care. Participants reported that the benefits of hearing aids were grossly inflated by marketing campaigns while their limitations were significantly minimised. This imbalance likely contributes to the formation of *unrealistic expectations for technology* (beliefs about consequences), specifically regarding hearing aids. Dissatisfaction is a known factor for the non-use of hearing aids, alongside challenges such as background noise (32).

The media was also criticised for failing to positively portray diverse hearing identities, which participants linked to *stigma* and general negative perceptions of hearing loss (social influence). These attitudes are well-documented barriers to hearing aid uptake (31, 33) and help-seeking behaviour (34–36). Conversely, social and peer support have been reported to be powerful facilitators to adult help-seeking behaviour and the successful adoption of hearing technology (37, 38).

Together, these interconnected themes reflect a landscape in which unhealthy hearing behaviours such as delayed or discontinued use of hearing loss interventions are perpetuated by informational, social, and structural barriers. These findings offer important guidance for the design of Hear4Health by acknowledging the overarching influence of knowledge and environmental context in shaping hearing behaviours. Therefore, to effectively influence healthy hearing behaviours, Hear4Health must deliver transparent, trustworthy, and consumer-centred education about both the benefits and limitations of hearing technologies and find opportunities to blend lived experience with evidence-based information. While communicating the proven advantages of hearing interventions will be pivotal for encouraging uptake and adoption (37), by setting realistic expectations through awareness of their limitations (e.g., noting that hearing aids may enhance speech understanding and reduce background noise, but cannot eliminate it entirely), Hear4Health can also endeavour to improve consumer satisfaction post-fitting and promote the continued use of hearing technologies for the best health outcomes (39–41).

### Holistic hearing healthcare

Another key observation from our findings was that the concept of holistic client-centred hearing healthcare underscored several themes and theoretical domains, including *poor awareness and education around hearing loss* (knowledge), *inadequacy of available hearing health information* (environmental context and resources), *informed decision making* (behaviour regulation), *self-efficacy* (beliefs about capabilities), and *value of lived experience* (social influence). In the context of our study, holistic hearing healthcare refers to support that extends beyond hearing aids and clinical interventions. It considers the broader impact of hearing loss on an individual, including their communication needs, lifestyle factors, emotional wellbeing, and social participation, and integrates a range of supports including assistive listening devices, communication strategies, environmental adaptations, peer support and community resources, mental health support, education and self-advocacy resources, and context-specific accommodations. A holistic approach to hearing healthcare has also been recommended by previous literature (42–44). However, in this current study, participants described a general lack of awareness of additional hearing supports within the community, which is compounded by the perceived absence of trustworthy, consumer-friendly hearing loss resources. These barriers ultimately contribute to poorer health outcomes and health behaviours among adults with hearing loss.

eHealth, or the use of online resources and digital tools to support healthcare, has been demonstrated to promote holistic hearing healthcare (45) by improving access to services, enhancing client outcomes and satisfaction (46–48), shortening delays in help-seeking (49), minimising hearing-related disability, and elevating psychosocial wellbeing (50). Leveraging our findings in the context of eHealth presents an opportunity to design our proposed online support platform, Hear4Health, in a way that optimises these outcomes. This comes at a time where internet-based platforms that deliver hearing loss-related education and resources are in growing demand (48).

Regarding holistic hearing healthcare, the domains of *knowledge* and *environmental context and resources* emerged from the interviews as the two primary opportunities to influence behaviour change in adults with hearing loss. A successful educational resource has been demonstrated to not only enhance health literacy and self-efficacy (47, 51), but also positively influence motivation and attitudes towards hearing loss management (52, 53). By increasing awareness of holistic hearing healthcare and providing access to high-quality hearing loss information on a comprehensive range of hearing supports, Hear4Health can empower consumers to make *informed care decisions* (behavioural regulation) and manage their hearing loss through improved health literacy and *self-efficacy* (beliefs about capabilities), both identified to be facilitators of healthier hearing behaviours and overall health outcomes in this study.

### Strengths and limitations

There were several strengths to this study. We were fortunate to draw on lived-experience perspectives in this study, not only from our consumers, but also from several Deafness Forum representatives as well as members of our research team who also have a hearing loss (AZ, LG). This ensured that lived-experienced insights informed all aspects of the study. Next, the use of semi-structured interviews enabled the interviewers to broach key topics consistently across participants while allowing each participant the space to elaborate on their unique experiences. This approach facilitated the collection of rich, nuanced data that could offer deeper insights into the barriers and facilitators of hearing healthcare access to better inform the design of the Hear4Health platform. Additionally, the application of the TDF provided a conceptual framework to guide qualitative data analysis (28). This framework offered a lens through which our qualitative findings could be interpreted into practical and actionable opportunities for Hear4Health to support healthy hearing behaviours.

One potential limitation of the study was that the interview guide was not initially developed with the TDF in mind but was instead informed by insights gathered during earlier exploratory focus groups. While Atkins et al. (28) recommended creating interview guides with open-ended questions mapped to each domain of the TDF, they also acknowledged that the number of domains addressed should be tailored to the specific target behaviour and available evidence (26). Therefore, it is difficult to determine whether all domains relevant to the research question were sufficiently addressed by the interview guide. However, since interview data were coded across all 14 TDF domains, the interview guide, derived from exploratory focus groups and further refined by the research and UX teams, was still able to facilitate the collection of comprehensive data covering a broad range of domains.

Another limitation of our study which may affect the generalisability of our results is the lack of culturally and linguistically diverse (CALD) participants. Only two participants were from non-Anglo-Saxon background, and all participants were fluent in English, as per our eligibility criteria. Existing literature indicates that while adults from ethnically-diverse backgrounds experience higher rates and greater risk of hearing loss (54, 55), intervention uptake is much lower than for the majority population (56, 57), due to a wide range of personal and environmental barriers (58). To ensure the accessibility and relevance of Hear4Health to a broad audience, it is essential to conduct additional research to identify the unique needs, challenges, and facilitators specific to CALD populations and incorporate these factors into the design of the platform.

## Conclusion

This study explored the barriers and facilitators influencing access to hearing loss information and interventions, with the aim of informing the design of the online hearing loss support service, Hear4Health. Identified barriers included low community awareness of hearing loss, limited access to trustworthy, effective information and resources for hearing loss, mistrust in the hearing industry driven by the overcommercialisation of hearing aids, stigma and negative social perceptions of hearing loss, unrealistic expectations for hearing technologies, and the complex dynamics underpinning deaf identity. Facilitators included peer and social support and the perceived value of lived experience, informed decision making, and self-efficacy of hearing loss management. These findings will directly shape the design of Hear4Health and highlight the need for (1) neutral, consumer-focused, evidence-based information and resources, (2) comprehensive coverage of the full range of hearing loss solutions and supports to promote holistic care, and (3) transparent descriptions of hearing technologies (such as hearing aids) outlining both their advantages and disadvantages. By equipping consumers with the relevant knowledge and skills, Hear4Health aims to build hearing health literacy and self-efficacy, enabling adults with hearing loss to successfully navigate their hearing healthcare and achieve improved health outcomes and quality of life.

## Data Availability

The raw data supporting the conclusions of this article will be made available by the authors, without undue reservation.
