# Supplementary material for: A qualitative study exploring access to online hearing loss information and support for adults with hearing loss
Source: Front Digit Health. 2026 Jun 23;8:1692717. doi: 10.3389/fdgth.2026.1692717 (PMC13337841; doi:10.3389/fdgth.2026.1692717)
Supplement: Supplementary file 1 [file Table1.docx]

**SUPPLEMENTARY FIGURES AND TABLES**

**Table 1.** Focus group agenda and guiding questions.

| **Agenda item** | **Guiding questions** |
| --- | --- |
| Discover consumer lived experiences with hearing loss and the hearing care pathway. | How did you access treatment to help you manage your hearing loss?  What information or resources would have helped you? |
| Explore existing experiences with online hearing health information and services. | **Goal**: identifying gaps, visibility of information and services.  What online **information** have you ***found*** for your hearing loss?   - Why were you looking for this information? - How did this information help you? - How often do you look for hearing health information?   What online **services** have you ***found*** for hearing loss?   - Why were you looking for this service? - How did this service help you? - How often do you access this service?   What additional online information or services could have been provided?   - How are you currently managing without this information? |
|  | **Goal**: discovering interactions with existing services.  What online **services** (web- or app-based) have you ***used*** for hearing health? |
| Show Australian Government Hearing Health media campaign videos. | **Goal**: assess the usability and accessibility of existing hearing health media.  Have you encountered these videos before?   - How did you find them? - Where did you find them?   Did it provide the information you wanted?  What did you like about the videos?  What did you not like about the videos?  What would you suggest to make these videos/campaigns more effective? |

**Table 2**. Semi-structured interview guide.

| **Section** | **Questions** |
| --- | --- |
| Pre-interview demographic collection | - Date of birth - Gender - Ethnicity - Country of birth - Highest level of education - Spoken languages - Use of AUSLAN - Employment status - Occupation |
| Lived experience | Can you tell me how you found out about your hearing loss?   - At what age did you suspect hearing loss?   How were you diagnosed with hearing loss?   - At what age were you diagnosed with hearing loss?   What treatment did you access for your hearing loss?   - When did you seek this treatment?   What information and resources were provided to you during your hearing loss treatment?   - What information would you have liked to receive during this process? - What did you feel was missing? |
| Online hearing health information | What kind of hearing health information do you access online?  How often do you access online hearing health resources?   - If not accessed, is this due to equity issues around device and connectivity availability? - Otherwise, what are some challenges you might have experienced in accessing online hearing health resources?   What information would be useful to you?   - Prompts: treatments, assistive technology, environmental adaptations, carer/family member information, NDIS and/or Government Hearing Services Program, support groups. |
| Public awareness of hearing health and hearing loss | How do you feel hearing health, hearing loss, and hearing services are portrayed in the media?   - How could this be improved? |
